# *N*,*N*′-Bis[2,6-bis­(1-methyl­eth­yl)phen­yl]pyridine-4-carboximidamide toluene hemisolvate

**DOI:** 10.1107/S2056989021000141

**Published:** 2021-01-12

**Authors:** Lola Cottin, Sarah Girard, Garry S. Hanan, Mihaela Cibian

**Affiliations:** a Département de Chimie, Biochimie et Physique et l’Institut de Recherche sur l’Hydrogène (IRH), Université du Québec à Trois-Rivières (UQTR), 3351, Boul. des Forges, C.P. 500, Trois-Rivières, QC, G9A 5H7, Canada; b Département de Chimie, Université de Montréal, campus MIL, 1375 Avenue, Thérèse-Lavoie-Roux, Montréal, QC, H2V 0B3, Canada

**Keywords:** crystal structure, bulky aryl­amidine, hydrogen bonding

## Abstract

The title compound, C_30_H_39_N_3_·0.5C_7_H_8_, is a symmetrically *N*,*N*′-disubstituted aryl­amidine containing a 4-pyridyl substituent on the carbon atom of the N–C–N linkage and bulky 2,6-diiso­propyl­phenyl groups on the nitro­gen atoms. It crystallizes in the *Z-anti* configuration and its amidine C—N bonds present amine [1.368 (1) Å] and imine [1.286 (1) Å] features. Intra­molecular hydrogen bonds are present in the structure together with inter­molecular N—H⋯N and C—H⋯N inter­actions linking the mol­ecules in chains along the *a-* and *c-*axis directions.

## Abstract 2

A new member of the bulky *N*,*N*′-bis­(2,6-diiso­propyl­phen­yl)aryl­amidine family is reported herein: the *N*,*N*′-bis­(2,6-diiso­propyl­phen­yl)-4-pyridyl­amidine is a symmetrically *N*,*N*′-disubstituted aryl­amidine containing a 4-pyridyl substituent on the carbon atom of the N–C–N linkage and bulky 2,6-diiso­propyl­phenyl groups on the nitro­gen atoms.

## Chemical context   

Amidine compounds are well developed in organic chemistry (Patai & Rappoport, 1991 ▸). Their derivatives are also good chelators for transition metals and their complexes have found widespread use in catalysis, polymerization reactions, as functional materials, and in supra­molecular chemisty (Bambirra *et al.*, 2004 ▸; Kazeminejad *et al.*, 2019 ▸; Qian *et al.*, 2010 ▸; Loh *et al.*, 2014 ▸; Boeré *et al.*, 1998 ▸; Chartrand & Hanan, 2008 ▸).
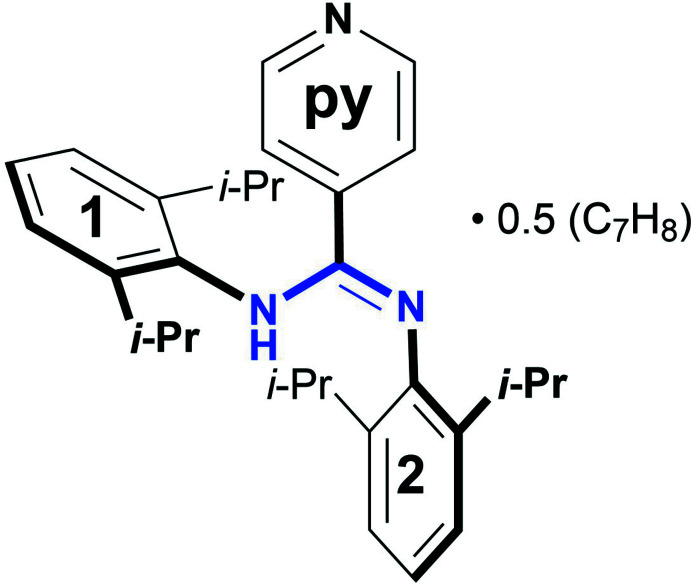


**Figure 1:**
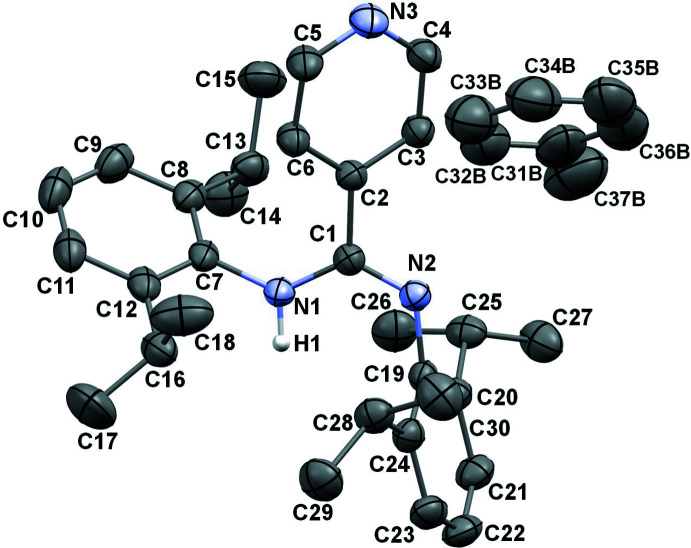
The mol­ecular structure of **1**, with displacement ellipsoids drawn at 50% probability level: main amidine moiety and co-crystallized toluene solvent (H atoms removed for clarity).

Herein, we report the synthesis and the solid state structure of *N,N*’-[2,6-bis­(1-methyl­eth­yl)phen­yl]-4-pyridine­carb­oxim­id­amide [*N*,*N*′-bis­(2,6-diiso­propyl­phen­yl)-4-pyridyl­amidine], which has been prepared as a potential ligand in coordination and supra­molecular chemistry and as precursor for the corresponding amidine-*N*-oxide derivative (Cibian *et al.*, 2011 ▸). For the specific example of the bulky *N*,*N*′-bis­(2,6-diiso­propyl­phen­yl)aryl­amidines, although crystallographic evidence of various of these compounds exists (Loh *et al.*, 2014 ▸; Boeré *et al.*, 1998 ▸), this is the first report of the 4-pyridyl-substituted compound (**1**) (Fig. 1 ▸).

## Structural commentary   

The mol­ecular structure of the title compound is illustrated in Fig. 1 ▸. A disordered toluene solvent (population of 0.5) is also present in the crystal structure. The amidine crystallizes completely in the *Z-anti* structure, the same as for *N*,*N*′-bis­(2,6-diiso­propyl­phen­yl)benzamidine (Loh *et al.*, 2014 ▸) and for *N*,*N*′-bis­(2,6-diiso­propyl­phen­yl)-4-anisyl­amidine (Boeré *et al.*, 1998 ▸), but differently from *N*,*N*′-bis­(2,6-diiso­propyl­phen­yl)-4-*t*Bu-benz­amidine (Jones *et al.*, 2011 ▸) and *N,N*’-bis­(2,6-diiso­propyl­phen­yl)-4-tolu­amidine (Boeré *et al.*, 1998 ▸) (which are disordered mixtures of *Z-anti* and *E-syn* tautomeric forms), as well as from *N*,*N*′-bis­(2,6-diiso­propyl­phen­yl)-acetamidine (entirely *E-anti*) (Boeré *et al.*, 1998 ▸).

The amidine C—N bonds in **1** present distinct amine [1.368 (1) Å] and imine [1.286 (1) Å] features, which is similar to what has been found in other bulky bis­(2,6-diiso­propyl­phen­yl)benzamidines that crystallized in only one isomeric/tautomeric form (Loh *et al.*, 2014 ▸; Boeré *et al.*, 1998 ▸).

The parameter *Δ*_CN_ = *d*(C—N) − *d*(C=N) for the central N–C–N amidine linkage (Häfelinger & Kuske, 1991 ▸) is generally used to assess the degree of delocalization in the N–C–N skeleton. In the title compound this difference is 0.082 (2) Å, whereas it is 0.081 (6) Å in *N*,*N*′-bis­(2,6-diiso­propyl­phen­yl)benzamidine (Loh *et al.*, 2014 ▸) and 0.057 (2) Å in *N*,*N*′-bis­(2,6-diiso­propyl­phen­yl)-4-anisyl­amidine (Boeré *et al.*, 1998 ▸). For non-substituted *N,N′*-di­phenyl­benzamidine, the same value of 0.057 Å (Alcock *et al.*, 1988 ▸) is found. As these are all compounds that crystallized in the *Z-anti* configuration only, the *Δ*_CN_ comparison indicates that although the substituents on the phenyl rings influence the degree of delocalization in the N–C–N amidine backbone, other factors also play an important role (*e.g.*, intra- and inter­molecular inter­actions and packing factors). It is important to note that for the compounds crystallized in mixtures of *Z-anti* and *E-syn* tautomeric forms, the value of *Δ*_CN_ is, as expected, significantly lower [*e.g.*, 0.019 (3) Å in *N*,*N*′-bis­(2,6-diiso­propyl­phen­yl)-4-*t*Bu-benzamidine (Jones *et al.*, 2011 ▸); 0.027 (4) Å in *N,N*’-bis­(2,6-diiso­propyl­phen­yl)-4-tolu­amidine (Boeré *et al.*, 1998 ▸)].

In the title compound, the pyridyl ring is tilted with respect to the central N–C–N bridge at an angle of 35.9 (1)°, while the bulky substituted aryl rings 1 and 2 (see scheme) are tilted by 65.2 (1) and 53.1 (1)°, respectively.

**Figure 2:**
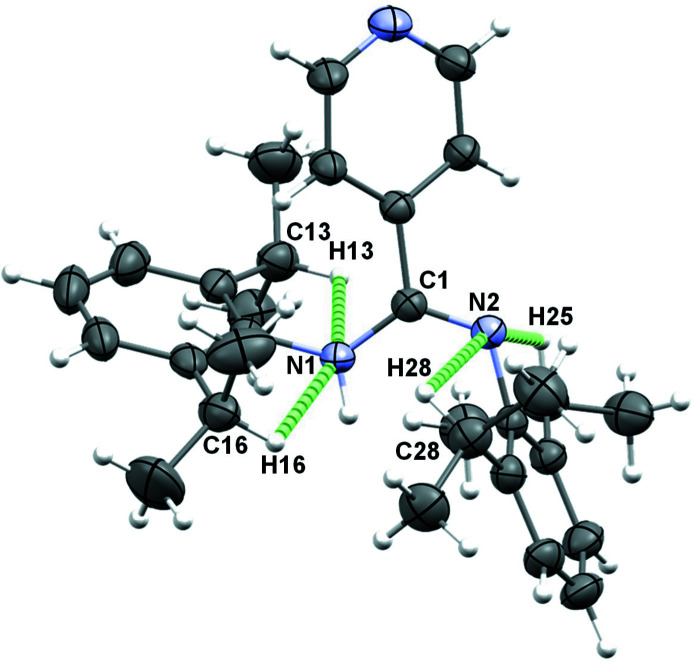
Intra­molecular hydrogen-bonding pattern in **1**. Co-crystallized solvent is omitted for clarity.

The intra­molecular hydrogen-bonding pattern in **1** (Table 1 ▸ and Fig. 2 ▸) reveals weak C—H⋯N hydrogen bonds (Desiraju & Steiner, 2001 ▸) between the (CH_3_)_2_C***H***– protons of each isopropyl substituent and the N atoms of the amidine bridge.

## Supra­molecular features   

**Figure 3:**
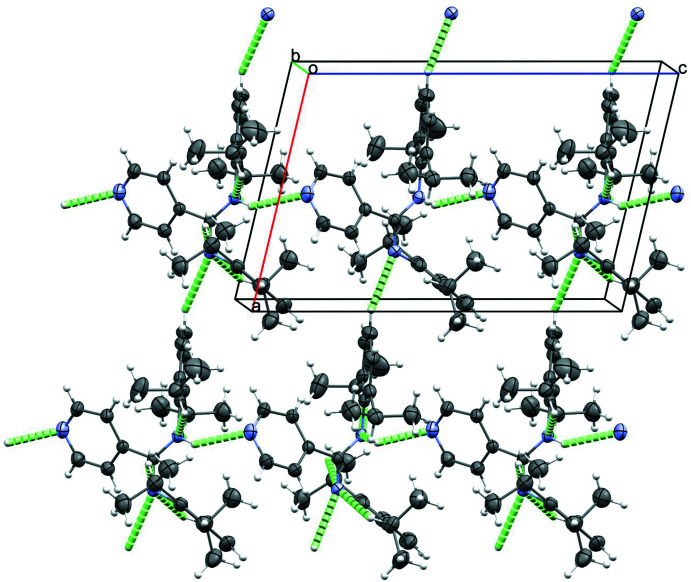
Inter­molecular hydrogen-bonding pattern in **1**. The mol­ecules are connected by N—H ⋯N and C—H⋯N inter­actions, forming infinite chains along the *a-* and *c-*axis directions.

In the crystal structure of **1**, two different types of conventional inter­molecular hydogen bonds (Table 1 ▸ and Fig. 3 ▸) (Desiraju & Steiner, 2001 ▸) can be identified, linking the discrete mol­ecules in infinite chains along the *a* and *c* axes. A relatively strong N—H ⋯N inter­action exists between the amidine H1 proton and the N3 pyridyl ring atom of an adjacent mol­ecule [angle N1—H1⋯N3 is 141 (1)°; distances H1⋯N3 and N1⋯N3 are 2.38 (1) and 3.118 (1) Å, respectively]. The second type of inter­molecular hydrogen bond is a much weaker C*sp* ^2^—H ⋯N inter­action between the *para* proton H10 of aryl ring 1 and the N2 amidine bridge atom of an adjacent mol­ecule [angle C10—H10 ⋯N2 is 139°; distances H10⋯N2 and C10⋯N3 are 2.74 Å and 3.515 (2) Å, respectively].

In the crystal packing, the chains of main amidine moieties (along the *a* axis) alternate with layers of co-crystallized toluene mol­ecules, but no real attractive inter­actions were identified between the main amidine and the toluene.

Furthermore, the packing analysis in **1** reveals two other inter­molecular short contacts of C*sp* ^2^—H ⋯π type [C4—H4 ⋯π (ring 2: C19–C24 aryl ring)] and C*sp* ^3^—H ⋯π type [C15—H15 ⋯π (pyridyl ring)] (Table 2 ▸), but no π–π type inter­actions. The formation of the latter is most probably hindered by the presence of the bulky 2,6-diisopropyl subs­tituents.

## Database survey   

Table 3 ▸ presents the results of the Cambridge Structural Database survey with respect to other reported mol­ecular structures of bulky *N*,*N*′-bis­(2,6-diiso­propyl­phen­yl)aryl­amidines (CSD version 5.41, update of May 2020; Groom *et al.*, 2016 ▸). All compounds reported in Table 3 ▸ are free bases non-coordinated to metals. Mol­ecular structures of coordination complexes of these ligands (as free base and depro­ton­ated forms) also exist [*e.g*., with molibdenum (GOBNAM; Boeré *et al.*, 1998 ▸); with lead (BAZVIJ; Jones *et al.*, 2011 ▸); with lithium, potassium, calcium (GIWGOK, GIWHAX, GIWHIF; Loh *et al.*, 2014 ▸); with magnesium (GIWLEF; Moxey *et al.*, 2014 ▸); with lanthanides (NAHDUW, NAHFEI, NAHFIM, NAHFUY; Bambirra *et al.*, 2004 ▸)]. In the case of *N*,*N*′-bis­(2,6-di-iso­propyl­phen­yl)-2,4,6-tri­methyl­benzamidine (Table 3 ▸, entry 6), the free-base ligand is co-crystallized with its coordin­ation complex (IKETAV; Green *et al.*, 2016 ▸). The compounds in Table 3 ▸ entries 1 to 6, are mono-amidines, while the compound in entry 7 is a phenyl-C-bridged bis-amidine (Li *et al.*, 2013 ▸). The solid-state structures of zirconium complexes with the 3,5-di-*t*-butyl-*N*,*N*′-bis­(2,6-di-iso­propyl­phen­yl)-2-oxybenzamidinato ligand also exist (CETCAH, CETCIP, CETCOV, CETDEM; Kirillov *et al.*, 2012 ▸), but the mol­ecular structure for the free-base non-coordinated form of this amidine has not yet been reported.

## Synthesis and crystallization   

***N*,*N*′** **-bis­[2,6-bis­(1-methyl­eth­yl)phen­yl]-4-pyridine­carb­ox­im­id­amide (1)**

Compound **1** was obtained from *N*-[2,6-bis­(1-methyl­eth­yl)phen­yl]-4-pyridine­carboxamide (Laramée *et al.*, 2012 ▸) and 2,6-diiso­propyl­aniline *via* the corresponding imidoyl chloride (Boeré *et al.*, 1998 ▸). *N*-[2,6-Bis(1-methyl­eth­yl)phen­yl]-4-pyridine­carboxamide (7.2 g, 25 mmol, 1 eq.), SOCl_2_ (30 mL, excess), dry Et_3_N (10 mL, 75 mmol, 3 eq.), 2,6-dii­propyl­aniline (5.3 mL, 28 mmol, 1.1 eq.), and dry toluene (50 mL) were combined following the general procedure for benzamidine synthesis reported in the above-mentioned reference. A beige precipitate was obtained directly from the reaction mixture, which was recrystallized in hot EtOH, to yield the desired product as a beige solid. X-ray quality crystals (colourless blocks) were obtained in EtOH/water (1:1) at 263 K. Yield 7.5 g, 66%. ^1^H NMR (DMSO-d_6_, 400 MHz) δ, ppm: 8.58–8.49 (*m*, 2H, **H**-py), 8.45 (*s*, 1H, **NH**), 7.57–7.49 (*m*, 1H, **H**-py), 7.41–7.33 (*m*, 1H, **H**-py), 7.33–7.25 (*m*, 1H, *p-* **H-**Ph), 7.23 (*d*, *J* = 8 Hz, 2H, *m-* **H-**Ph), 6.87 (*d*, *J* = 8 Hz, 2H, *m-* **H-**Ph), 6.83–6.76 (*m*, 1H, *p-* **H-**Ph), 3.43 [*sept*, *J* = 7 Hz, 2H, –**CH**–(CH_3_)_2_], 2.99 [*sept*, *J* = 7 Hz, 2H, –**CH**–(CH_3_)_2_], 1.30 [*d*, *J* = 7 Hz, 6H, –CH–**(CH_3_)_2_**), 1.24 (*d, J* = 7 Hz, 6H, –CH–**(CH_3_)_2_**), 0.91 (*d, J* = 7 Hz 6H, –CH–**(CH_3_)_2_**], 0.80 [*d, J* = 7 Hz, 6H, –CH–**(CH_3_)_2_**].

## Refinement   

Crystal data, data collection and structure refinement details are summarized in Table 4 ▸. H atoms were included in calculated positions and treated as riding atoms: aromatic C—H 0.95 Å, methyl C—H 0.98 Å, with Uiso(H) = *k* × *U*eq(parent C-atom), where *k* = 1.2 for the aromatic H atoms and 1.5 for the methyl H atoms. The NH proton (H1) was located in the difference-Fourier map and refined freely.

Co-crystallized disordered solvent (toluene, which was the reaction solvent) present on a symmetry position was modelled as two component disorder using PART −1 and PART −2 instructions. The occupancy factor was fixed at 0.25. The following constraints and restraints were also used: DFIX, FLAT and SADI (on position), ISOR and SIMU (on thermal factors). The model was refined anisotropically.

## Supplementary Material

Crystal structure: contains datablock(s) I. DOI: 10.1107/S2056989021000141/dj2016sup1.cif

Structure factors: contains datablock(s) I. DOI: 10.1107/S2056989021000141/dj2016Isup2.hkl

CCDC reference: 2054114

Additional supporting information:  crystallographic information; 3D view; checkCIF report

## Figures and Tables

**Table 1:** Hydrogen-bond geometry (Å, °)

*D*—H⋯*A*	*D*—H	H⋯*A*	*D*⋯*A*	*D*—H⋯*A*
N1—H1⋯N3^i^	0.89 (1)	2.38 (1)	3.118 (1)	141 (1)
C10—H10⋯N2^ii^	0.95	2.74	3.515 (2)	139
C13—H13⋯N1	1.00	2.50	2.9794 (15)	109
C16—H16⋯N1	1.00	2.44	2.8811 (15)	106
C25—H25⋯N2	1.00	2.42	2.8933 (15)	108
C28—H28⋯N2	1.00	2.54	2.9140 (15)	102

Symmetry codes: (i) 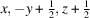; (ii) 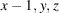.

**Table 2:** Inter­molecular short contacts in **1** (Å, °) *Cg* (py) is the centroid of the pyridyl ring. *Cg* (ring 2) is the centroid of the C19–C24 aryl ring.

*X*—H⋯*Cg* (π-ring)	H⋯*Cg*	*X*⋯*Cg*	*X*—H⋯*Cg*
C4—H4⋯*Cg* (ring 2)^iii^	2.88	3.53 (1)	127
C15—H15⋯*Cg* (py)^iv^	2.82	3.71 (1)	151

Symmetry codes: (iii) *x*, 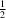 − *y*, −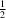 + *z*; (iv) *x*, *y*, *z*.

**Table 3:** CSD reported mol­ecular structures of bulky *N*,*N*′-bis­(2,6-diiso­propyl­phen­yl)**ar­yl**amidines (free-base non-coordinated forms)

No.	Aryl substituent	CSD refcode	Reference
1	Ph	GIWGEA	Loh *et al.* (2014 ▸)
2	4-MePh	GOBNIU	Boeré *et al.* (1998 ▸)
3	4-OMePh	GOBMOZ	Boeré *et al.* (1998 ▸)
4	4 − *t*-BuPh	BAZTUT	Jones *et al.* (2011 ▸)
5	3,5-diMePh	GIWLEF	Moxey *et al.* (2014 ▸)
6	2,4,6-triMePh	IKETAV	Green *et al.* (2016 ▸)
7	Ph (C-bridged)	DIFCIG	Li *et al.* (2013 ▸)

**Table 4:** Experimental details

Crystal data	
Chemical formula	2C_30_H_39_N_3_·C_7_H_8_
*M*_r_	975.41
Crystal system, space group	Monoclinic, *P*2_1_/*c*
Temperature (K)	200
*a*, *b*, *c* (Å)	9.7537 (2), 20.8030 (5), 14.7561 (4)
β (°)	103.422 (1)
*V* (Å^3^)	2912.33 (12)
*Z*	2
Radiation type	Cu *K*α
μ (mm^−1^)	0.49
Crystal size (mm)	0.32 × 0.12 × 0.12

Data collection	
Diffractometer	Bruker APEXII CCD
Absorption correction	Multi-scan (*SADABS*; Bruker, 2014 ▸/4)
*T*_min_, *T*_max_	0.629, 0.754
No. of measured, independent and observed [*I* > 2σ(*I*)] reflections	36670, 5649, 4950
*R*_int_	0.035
(sin θ/λ)_max_ (Å^−1^)	0.618

Refinement	
*R*[*F* ^2^ > 2σ(*F* ^2^)], *wR*(*F* ^2^), *S*	0.043, 0.120, 1.05
No. of reflections	5649
No. of parameters	424
No. of restraints	433
H-atom treatment	H atoms treated by a mixture of independent and constrained refinement
Δρ_max_, Δρ_min_ (e Å^−3^)	0.26, −0.20

Computer programs: *APEX2* and *SAINT* (Bruker, 2009 ▸), *SHELXT* (Sheldrick, 2015*a* ▸), *SHELXL* (Sheldrick, 2015*b* ▸), *OLEX2* (Dolomanov *et al.*, 2009 ▸), *ORTEP-3 for Windows* (Farrugia, 2012 ▸), pubCIF (Westrip, 2010 ▸), *POV-RAY* (Povray, 2013 ▸), *PLATON* (Spek, 2020 ▸) and *Mercury* (Macrae *et al.*, 2020 ▸).

## supplementary crystallographic information

### Crystal data

**Table:**

2C_30_H_39_N_3_·C_7_H_8_	*F*(000) = 1060
*M**_r_* = 975.41	*D*_x_ = 1.112 Mg m^−^^3^
Monoclinic, *P*2_1_/*c*	Cu *K*α radiation, λ = 1.54178 Å
*a* = 9.7537 (2) Å	Cell parameters from 9920 reflections
*b* = 20.8030 (5) Å	θ = 3.7–71.8°
*c* = 14.7561 (4) Å	µ = 0.49 mm^−^^1^
β = 103.422 (1)°	*T* = 200 K
*V* = 2912.33 (12) Å^3^	Block, colourless
*Z* = 2	0.32 × 0.12 × 0.12 mm

### Data collection

**Table:**

Bruker APEXII CCD diffractometer	5649 independent reflections
Radiation source: rotating-anode with a mirror focussing unit	4950 reflections with *I* > 2σ(*I*)
Graphite monochromator	*R*_int_ = 0.035
φ and ω scans	θ_max_ = 72.4°, θ_min_ = 3.7°
Absorption correction: multi-scan (SADABS; Bruker, 2014/4)	*h* = −11→11
*T*_min_ = 0.629, *T*_max_ = 0.754	*k* = −25→25
36670 measured reflections	*l* = −15→17

### Refinement

**Table:**

Refinement on *F*^2^	Hydrogen site location: mixed
Least-squares matrix: full	H atoms treated by a mixture of independent and constrained refinement
*R*[*F*^2^ > 2σ(*F*^2^)] = 0.043	*w* = 1/[σ^2^(*F*_o_^2^) + (0.0682*P*)^2^ + 0.4597*P*] where *P* = (*F*_o_^2^ + 2*F*_c_^2^)/3
*wR*(*F*^2^) = 0.120	(Δ/σ)_max_ < 0.001
*S* = 1.05	Δρ_max_ = 0.26 e Å^−^^3^
5649 reflections	Δρ_min_ = −0.20 e Å^−^^3^
424 parameters	Extinction correction: SHELXL, Fc^*^=kFc[1+0.001xFc^2^λ^3^/sin(2θ)]^-1/4^
433 restraints	Extinction coefficient: 0.0058 (3)
Primary atom site location: dual	

### Special details

**Table:**

Experimental. X-ray crystallographic data for I were collected from a single-crystal sample, which was mounted on a loop fiber. Data were collected using a Bruker Platform diffractometer, equipped with a Bruker *SMART* 4 K Charged-Coupled Device (CCD) Area Detector using the program *APEX2* and a Nonius FR591 rotating anode equiped with a Montel 200 optics. The crystal-to-detector distance was 5.0 cm, and the data collection was carried out in 512 *x* 512 pixel mode. The initial unit-cell parameters were determined by a least-squares fit of the angular settings of strong reflections, collected by a 10.0 degree scan in 33 frames over four different parts of the reciprocal space (132 frames total). One complete sphere of data was collected, to better than 0.80Å resolution.
Geometry. All esds (except the esd in the dihedral angle between two l.s. planes) are estimated using the full covariance matrix. The cell esds are taken into account individually in the estimation of esds in distances, angles and torsion angles; correlations between esds in cell parameters are only used when they are defined by crystal symmetry. An approximate (isotropic) treatment of cell esds is used for estimating esds involving l.s. planes.

### Fractional atomic coordinates and isotropic or equivalent isotropic displacement parameters (Å^2^)

**Table:**

	*x*	*y*	*z*	*U*_iso_*/*U*_eq_	Occ. (<1)
N1	0.53487 (9)	0.28098 (4)	0.39210 (6)	0.0314 (2)	
N2	0.76154 (9)	0.28020 (4)	0.36187 (6)	0.0303 (2)	
N3	0.51539 (11)	0.15891 (5)	0.08225 (7)	0.0432 (3)	
C1	0.63027 (10)	0.26479 (5)	0.34104 (7)	0.0278 (2)	
C2	0.58249 (11)	0.22664 (5)	0.25252 (7)	0.0293 (2)	
C3	0.67636 (11)	0.18300 (5)	0.22828 (8)	0.0347 (2)	
H3	0.7655	0.1754	0.2693	0.042*	
C4	0.63893 (13)	0.15082 (6)	0.14405 (8)	0.0403 (3)	
H4	0.7046	0.1212	0.1291	0.048*	
C5	0.42520 (13)	0.20012 (6)	0.10671 (8)	0.0412 (3)	
H5	0.3364	0.2063	0.0646	0.049*	
C6	0.45284 (11)	0.23457 (6)	0.18962 (7)	0.0353 (2)	
H6	0.3843	0.2631	0.2033	0.042*	
C7	0.38815 (11)	0.26451 (5)	0.37550 (7)	0.0325 (2)	
C8	0.34635 (12)	0.20029 (6)	0.38124 (8)	0.0380 (3)	
C9	0.20106 (14)	0.18795 (7)	0.36374 (9)	0.0500 (3)	
H9	0.1695	0.1448	0.3649	0.060*	
C10	0.10303 (13)	0.23682 (8)	0.34497 (10)	0.0542 (4)	
H10	0.0053	0.2271	0.3332	0.065*	
C11	0.14653 (13)	0.29971 (7)	0.34320 (9)	0.0478 (3)	
H11	0.0783	0.3331	0.3321	0.057*	
C12	0.28919 (12)	0.31517 (6)	0.35745 (7)	0.0379 (3)	
C13	0.44931 (14)	0.14485 (6)	0.40914 (9)	0.0433 (3)	
H13	0.5466	0.1617	0.4131	0.052*	
C14	0.44272 (17)	0.11946 (7)	0.50602 (10)	0.0567 (4)	
H14A	0.4637	0.1545	0.5515	0.085*	
H14B	0.5122	0.0850	0.5244	0.085*	
H14C	0.3481	0.1027	0.5038	0.085*	
C15	0.42194 (18)	0.09041 (7)	0.33691 (12)	0.0610 (4)	
H15A	0.3273	0.0728	0.3322	0.092*	
H15B	0.4921	0.0564	0.3563	0.092*	
H15C	0.4290	0.1073	0.2761	0.092*	
C16	0.33464 (13)	0.38487 (6)	0.35151 (9)	0.0440 (3)	
H16	0.4341	0.3883	0.3886	0.053*	
C17	0.2473 (2)	0.43221 (10)	0.39327 (18)	0.0935 (7)	
H17A	0.1518	0.4346	0.3535	0.140*	
H17B	0.2912	0.4748	0.3975	0.140*	
H17C	0.2426	0.4177	0.4557	0.140*	
C18	0.3345 (2)	0.40341 (8)	0.25163 (11)	0.0725 (5)	
H18A	0.3964	0.3742	0.2274	0.109*	
H18B	0.3685	0.4476	0.2503	0.109*	
H18C	0.2384	0.4003	0.2129	0.109*	
C19	0.82379 (10)	0.31763 (5)	0.44194 (7)	0.0299 (2)	
C20	0.90785 (11)	0.28642 (5)	0.52053 (8)	0.0340 (2)	
C21	0.98604 (13)	0.32403 (6)	0.59266 (8)	0.0423 (3)	
H21	1.0435	0.3036	0.6457	0.051*	
C22	0.98145 (13)	0.39049 (6)	0.58836 (9)	0.0457 (3)	
H22	1.0365	0.4153	0.6376	0.055*	
C23	0.89641 (13)	0.42074 (6)	0.51204 (9)	0.0416 (3)	
H23	0.8926	0.4664	0.5101	0.050*	
C24	0.81617 (11)	0.38542 (5)	0.43795 (8)	0.0346 (3)	
C25	0.90836 (13)	0.21355 (6)	0.52829 (8)	0.0398 (3)	
H25	0.8783	0.1956	0.4638	0.048*	
C26	0.79988 (16)	0.19247 (6)	0.58252 (11)	0.0531 (3)	
H26A	0.8285	0.2082	0.6467	0.080*	
H26B	0.7944	0.1454	0.5828	0.080*	
H26C	0.7074	0.2103	0.5527	0.080*	
C27	1.05315 (15)	0.18549 (7)	0.57291 (10)	0.0542 (4)	
H27A	1.1228	0.2017	0.5402	0.081*	
H27B	1.0490	0.1385	0.5686	0.081*	
H27C	1.0804	0.1983	0.6385	0.081*	
C28	0.72676 (13)	0.41935 (6)	0.35301 (9)	0.0411 (3)	
H28	0.6473	0.3899	0.3247	0.049*	
C29	0.66220 (16)	0.48254 (6)	0.37642 (11)	0.0559 (4)	
H29A	0.6139	0.4752	0.4268	0.084*	
H29B	0.5944	0.4983	0.3211	0.084*	
H29C	0.7370	0.5145	0.3963	0.084*	
C30	0.81250 (17)	0.43109 (7)	0.27984 (10)	0.0579 (4)	
H30A	0.8904	0.4606	0.3051	0.087*	
H30B	0.7516	0.4500	0.2240	0.087*	
H30C	0.8504	0.3902	0.2636	0.087*	
C37B	1.0818 (16)	−0.0445 (6)	0.6520 (6)	0.112 (4)	0.25
H37A	1.0328	−0.0847	0.6587	0.168*	0.25
H37B	1.0700	−0.0142	0.7004	0.168*	0.25
H37C	1.1823	−0.0532	0.6583	0.168*	0.25
C31B	1.0211 (9)	−0.0160 (4)	0.5579 (5)	0.080 (3)	0.25
C32B	0.8827 (9)	0.0063 (6)	0.5345 (6)	0.079 (4)	0.25
H32B	0.8254	0.0024	0.5782	0.095*	0.25
C33B	0.8272 (9)	0.0339 (6)	0.4482 (7)	0.084 (4)	0.25
H33B	0.7326	0.0490	0.4334	0.101*	0.25
C34B	0.9093 (13)	0.0397 (4)	0.3836 (5)	0.088 (4)	0.25
H34B	0.8715	0.0587	0.3245	0.105*	0.25
C35B	1.0473 (13)	0.0176 (4)	0.4059 (6)	0.093 (4)	0.25
H35B	1.1043	0.0215	0.3620	0.111*	0.25
C36B	1.1022 (8)	−0.0100 (4)	0.4922 (7)	0.090 (4)	0.25
H36B	1.1966	−0.0251	0.5067	0.109*	0.25
C31A	1.0600 (10)	−0.0150 (4)	0.5303 (7)	0.065 (2)	0.25
C32A	0.9297 (14)	−0.0029 (7)	0.5489 (10)	0.070 (4)	0.25
H32A	0.9145	−0.0139	0.6083	0.084*	0.25
C33A	0.8199 (12)	0.0251 (6)	0.4829 (10)	0.080 (3)	0.25
H33A	0.7296	0.0310	0.4957	0.096*	0.25
C34A	0.8457 (13)	0.0441 (11)	0.3983 (11)	0.073 (3)	0.25
H34A	0.7763	0.0675	0.3549	0.088*	0.25
C35A	0.9736 (11)	0.0288 (6)	0.3772 (8)	0.074 (3)	0.25
H35A	0.9873	0.0379	0.3168	0.089*	0.25
C36A	1.0805 (13)	0.0008 (8)	0.4423 (9)	0.060 (3)	0.25
H36A	1.1685	−0.0078	0.4276	0.072*	0.25
C37A	1.1873 (16)	−0.0441 (10)	0.5950 (11)	0.082 (4)	0.25
H37D	1.1802	−0.0383	0.6596	0.122*	0.25
H37E	1.2727	−0.0228	0.5858	0.122*	0.25
H37F	1.1918	−0.0901	0.5816	0.122*	0.25
H1	0.5679 (14)	0.3064 (6)	0.4406 (10)	0.039 (3)*	

### Atomic displacement parameters (Å^2^)

**Table:**

	*U*^11^	*U*^22^	*U*^33^	*U*^12^	*U*^13^	*U*^23^
N1	0.0291 (5)	0.0388 (5)	0.0266 (5)	−0.0033 (4)	0.0069 (3)	−0.0071 (4)
N2	0.0285 (4)	0.0331 (4)	0.0289 (5)	−0.0007 (3)	0.0057 (3)	−0.0012 (3)
N3	0.0457 (6)	0.0542 (6)	0.0296 (5)	0.0007 (5)	0.0082 (4)	−0.0078 (4)
C1	0.0294 (5)	0.0285 (5)	0.0252 (5)	0.0016 (4)	0.0059 (4)	0.0027 (4)
C2	0.0317 (5)	0.0325 (5)	0.0245 (5)	−0.0026 (4)	0.0080 (4)	0.0004 (4)
C3	0.0322 (5)	0.0388 (6)	0.0326 (6)	0.0013 (4)	0.0065 (4)	−0.0017 (4)
C4	0.0422 (6)	0.0438 (6)	0.0363 (6)	0.0041 (5)	0.0115 (5)	−0.0060 (5)
C5	0.0383 (6)	0.0571 (7)	0.0265 (6)	0.0023 (5)	0.0037 (4)	−0.0034 (5)
C6	0.0336 (6)	0.0451 (6)	0.0271 (5)	0.0037 (4)	0.0072 (4)	−0.0010 (4)
C7	0.0296 (5)	0.0462 (6)	0.0229 (5)	−0.0028 (4)	0.0082 (4)	−0.0051 (4)
C8	0.0370 (6)	0.0496 (6)	0.0292 (5)	−0.0080 (5)	0.0114 (4)	−0.0053 (5)
C9	0.0429 (7)	0.0655 (8)	0.0437 (7)	−0.0177 (6)	0.0142 (5)	−0.0037 (6)
C10	0.0312 (6)	0.0890 (11)	0.0433 (7)	−0.0083 (6)	0.0106 (5)	0.0000 (7)
C11	0.0331 (6)	0.0740 (9)	0.0369 (6)	0.0069 (6)	0.0091 (5)	−0.0010 (6)
C12	0.0345 (6)	0.0552 (7)	0.0241 (5)	0.0042 (5)	0.0071 (4)	−0.0044 (5)
C13	0.0475 (7)	0.0395 (6)	0.0456 (7)	−0.0094 (5)	0.0160 (5)	−0.0042 (5)
C14	0.0642 (9)	0.0534 (8)	0.0544 (8)	−0.0059 (7)	0.0180 (7)	0.0078 (6)
C15	0.0736 (10)	0.0466 (7)	0.0666 (10)	−0.0133 (7)	0.0239 (8)	−0.0155 (7)
C16	0.0413 (6)	0.0484 (7)	0.0390 (6)	0.0090 (5)	0.0023 (5)	−0.0063 (5)
C17	0.0892 (14)	0.0726 (12)	0.1265 (18)	0.0115 (10)	0.0410 (13)	−0.0375 (12)
C18	0.1071 (14)	0.0545 (9)	0.0468 (8)	−0.0115 (9)	−0.0007 (8)	0.0049 (7)
C19	0.0257 (5)	0.0352 (5)	0.0295 (5)	−0.0031 (4)	0.0076 (4)	−0.0018 (4)
C20	0.0319 (5)	0.0389 (6)	0.0309 (6)	−0.0016 (4)	0.0065 (4)	−0.0009 (4)
C21	0.0399 (6)	0.0505 (7)	0.0328 (6)	−0.0050 (5)	0.0010 (5)	−0.0018 (5)
C22	0.0445 (7)	0.0504 (7)	0.0400 (7)	−0.0155 (5)	0.0049 (5)	−0.0109 (5)
C23	0.0441 (6)	0.0363 (6)	0.0461 (7)	−0.0107 (5)	0.0138 (5)	−0.0054 (5)
C24	0.0332 (5)	0.0355 (6)	0.0365 (6)	−0.0047 (4)	0.0110 (4)	−0.0007 (4)
C25	0.0465 (7)	0.0383 (6)	0.0304 (6)	0.0029 (5)	0.0008 (5)	0.0006 (4)
C26	0.0625 (8)	0.0405 (7)	0.0567 (8)	−0.0030 (6)	0.0148 (7)	0.0080 (6)
C27	0.0578 (8)	0.0549 (8)	0.0446 (7)	0.0164 (6)	0.0010 (6)	−0.0003 (6)
C28	0.0449 (6)	0.0337 (6)	0.0433 (7)	−0.0008 (5)	0.0073 (5)	0.0031 (5)
C29	0.0612 (9)	0.0417 (7)	0.0640 (9)	0.0081 (6)	0.0128 (7)	0.0031 (6)
C30	0.0705 (9)	0.0582 (8)	0.0474 (8)	0.0082 (7)	0.0183 (7)	0.0119 (6)
C37B	0.105 (10)	0.081 (8)	0.134 (9)	−0.015 (7)	−0.003 (7)	−0.007 (6)
C31B	0.071 (6)	0.049 (4)	0.120 (8)	−0.020 (5)	0.020 (5)	−0.014 (6)
C32B	0.077 (7)	0.071 (8)	0.091 (7)	−0.012 (5)	0.023 (5)	−0.017 (5)
C33B	0.090 (7)	0.084 (8)	0.081 (7)	−0.010 (6)	0.026 (5)	−0.010 (6)
C34B	0.103 (9)	0.083 (8)	0.083 (6)	−0.017 (7)	0.034 (6)	−0.022 (5)
C35B	0.097 (8)	0.061 (7)	0.131 (10)	−0.011 (6)	0.048 (6)	−0.013 (6)
C36B	0.087 (7)	0.057 (7)	0.135 (10)	−0.013 (6)	0.042 (6)	−0.013 (7)
C31A	0.079 (5)	0.034 (3)	0.089 (5)	−0.013 (4)	0.036 (3)	−0.016 (3)
C32A	0.072 (6)	0.055 (9)	0.094 (7)	−0.002 (5)	0.042 (5)	−0.006 (6)
C33A	0.078 (5)	0.049 (5)	0.112 (7)	−0.006 (4)	0.023 (5)	−0.007 (5)
C34A	0.049 (5)	0.064 (6)	0.106 (8)	0.004 (5)	0.016 (4)	−0.011 (5)
C35A	0.067 (6)	0.061 (6)	0.095 (6)	−0.013 (5)	0.017 (5)	−0.013 (4)
C36A	0.056 (5)	0.048 (8)	0.081 (6)	−0.005 (5)	0.029 (4)	−0.013 (5)
C37A	0.094 (8)	0.061 (7)	0.092 (8)	−0.002 (7)	0.025 (6)	−0.014 (6)

### Geometric parameters (Å, º)

**Table:**

N1—C1	1.3682 (13)	C22—C23	1.3851 (18)
N1—C7	1.4362 (13)	C23—H23	0.9500
N1—H1	0.888 (14)	C23—C24	1.3966 (16)
N2—C1	1.2861 (13)	C24—C28	1.5239 (16)
N2—C19	1.4275 (13)	C25—H25	1.0000
N3—C4	1.3423 (16)	C25—C26	1.5314 (19)
N3—C5	1.3367 (16)	C25—C27	1.5286 (17)
C1—C2	1.5069 (14)	C26—H26A	0.9800
C2—C3	1.3938 (15)	C26—H26B	0.9800
C2—C6	1.3939 (15)	C26—H26C	0.9800
C3—H3	0.9500	C27—H27A	0.9800
C3—C4	1.3841 (16)	C27—H27B	0.9800
C4—H4	0.9500	C27—H27C	0.9800
C5—H5	0.9500	C28—H28	1.0000
C5—C6	1.3891 (16)	C28—C29	1.5312 (18)
C6—H6	0.9500	C28—C30	1.5311 (19)
C7—C8	1.4051 (16)	C29—H29A	0.9800
C7—C12	1.4120 (16)	C29—H29B	0.9800
C8—C9	1.4039 (17)	C29—H29C	0.9800
C8—C13	1.5218 (18)	C30—H30A	0.9800
C9—H9	0.9500	C30—H30B	0.9800
C9—C10	1.379 (2)	C30—H30C	0.9800
C10—H10	0.9500	C37B—H37A	0.9800
C10—C11	1.377 (2)	C37B—H37B	0.9800
C11—H11	0.9500	C37B—H37C	0.9800
C11—C12	1.3957 (17)	C37B—C31B	1.4987
C12—C16	1.5249 (18)	C31B—C32B	1.3924
C13—H13	1.0000	C31B—C36B	1.3918
C13—C14	1.5394 (19)	C32B—H32B	0.9500
C13—C15	1.5356 (18)	C32B—C33B	1.3872
C14—H14A	0.9800	C33B—H33B	0.9500
C14—H14B	0.9800	C33B—C34B	1.3864
C14—H14C	0.9800	C34B—H34B	0.9500
C15—H15A	0.9800	C34B—C35B	1.3871
C15—H15B	0.9800	C35B—H35B	0.9500
C15—H15C	0.9800	C35B—C36B	1.3872
C16—H16	1.0000	C36B—H36B	0.9500
C16—C17	1.523 (2)	C31A—C32A	1.384 (10)
C16—C18	1.523 (2)	C31A—C36A	1.398 (10)
C17—H17A	0.9800	C31A—C37A	1.506 (11)
C17—H17B	0.9800	C32A—H32A	0.9500
C17—H17C	0.9800	C32A—C33A	1.397 (10)
C18—H18A	0.9800	C33A—H33A	0.9500
C18—H18B	0.9800	C33A—C34A	1.388 (10)
C18—H18C	0.9800	C34A—H34A	0.9500
C19—C20	1.4133 (15)	C34A—C35A	1.391 (10)
C19—C24	1.4127 (15)	C35A—H35A	0.9500
C20—C21	1.3963 (16)	C35A—C36A	1.373 (10)
C20—C25	1.5203 (16)	C36A—H36A	0.9500
C21—H21	0.9500	C37A—H37D	0.9800
C21—C22	1.3843 (19)	C37A—H37E	0.9800
C22—H22	0.9500	C37A—H37F	0.9800
			
C1—N1—C7	128.72 (9)	C22—C23—C24	121.23 (11)
C1—N1—H1	115.1 (9)	C24—C23—H23	119.4
C7—N1—H1	116.1 (9)	C19—C24—C28	120.75 (10)
C1—N2—C19	122.84 (9)	C23—C24—C19	118.56 (10)
C5—N3—C4	116.11 (10)	C23—C24—C28	120.66 (10)
N1—C1—C2	119.60 (9)	C20—C25—H25	107.7
N2—C1—N1	125.01 (9)	C20—C25—C26	109.53 (10)
N2—C1—C2	115.39 (9)	C20—C25—C27	113.56 (10)
C3—C2—C1	118.44 (9)	C26—C25—H25	107.7
C3—C2—C6	117.04 (10)	C27—C25—H25	107.7
C6—C2—C1	124.41 (9)	C27—C25—C26	110.30 (11)
C2—C3—H3	120.2	C25—C26—H26A	109.5
C4—C3—C2	119.57 (10)	C25—C26—H26B	109.5
C4—C3—H3	120.2	C25—C26—H26C	109.5
N3—C4—C3	123.89 (11)	H26A—C26—H26B	109.5
N3—C4—H4	118.1	H26A—C26—H26C	109.5
C3—C4—H4	118.1	H26B—C26—H26C	109.5
N3—C5—H5	117.9	C25—C27—H27A	109.5
N3—C5—C6	124.29 (11)	C25—C27—H27B	109.5
C6—C5—H5	117.9	C25—C27—H27C	109.5
C2—C6—H6	120.5	H27A—C27—H27B	109.5
C5—C6—C2	119.08 (10)	H27A—C27—H27C	109.5
C5—C6—H6	120.5	H27B—C27—H27C	109.5
C8—C7—N1	120.54 (10)	C24—C28—H28	107.5
C8—C7—C12	121.72 (10)	C24—C28—C29	113.51 (11)
C12—C7—N1	117.69 (10)	C24—C28—C30	110.60 (10)
C7—C8—C13	123.67 (10)	C29—C28—H28	107.5
C9—C8—C7	117.22 (12)	C30—C28—H28	107.5
C9—C8—C13	119.06 (11)	C30—C28—C29	110.05 (11)
C8—C9—H9	119.2	C28—C29—H29A	109.5
C10—C9—C8	121.69 (13)	C28—C29—H29B	109.5
C10—C9—H9	119.2	C28—C29—H29C	109.5
C9—C10—H10	119.9	H29A—C29—H29B	109.5
C11—C10—C9	120.12 (12)	H29A—C29—H29C	109.5
C11—C10—H10	119.9	H29B—C29—H29C	109.5
C10—C11—H11	119.4	C28—C30—H30A	109.5
C10—C11—C12	121.11 (13)	C28—C30—H30B	109.5
C12—C11—H11	119.4	C28—C30—H30C	109.5
C7—C12—C16	121.70 (10)	H30A—C30—H30B	109.5
C11—C12—C7	118.06 (12)	H30A—C30—H30C	109.5
C11—C12—C16	120.23 (11)	H30B—C30—H30C	109.5
C8—C13—H13	108.0	H37A—C37B—H37B	109.5
C8—C13—C14	110.13 (11)	H37A—C37B—H37C	109.5
C8—C13—C15	111.95 (11)	H37B—C37B—H37C	109.5
C14—C13—H13	108.0	C31B—C37B—H37A	109.5
C15—C13—H13	108.0	C31B—C37B—H37B	109.5
C15—C13—C14	110.70 (11)	C31B—C37B—H37C	109.5
C13—C14—H14A	109.5	C32B—C31B—C37B	120.9
C13—C14—H14B	109.5	C36B—C31B—C37B	120.9
C13—C14—H14C	109.5	C36B—C31B—C32B	118.1
H14A—C14—H14B	109.5	C31B—C32B—H32B	119.5
H14A—C14—H14C	109.5	C33B—C32B—C31B	121.1
H14B—C14—H14C	109.5	C33B—C32B—H32B	119.5
C13—C15—H15A	109.5	C32B—C33B—H33B	119.9
C13—C15—H15B	109.5	C34B—C33B—C32B	120.1
C13—C15—H15C	109.5	C34B—C33B—H33B	119.9
H15A—C15—H15B	109.5	C33B—C34B—H34B	120.3
H15A—C15—H15C	109.5	C33B—C34B—C35B	119.5
H15B—C15—H15C	109.5	C35B—C34B—H34B	120.3
C12—C16—H16	107.1	C34B—C35B—H35B	119.9
C17—C16—C12	113.21 (13)	C34B—C35B—C36B	120.1
C17—C16—H16	107.1	C36B—C35B—H35B	119.9
C18—C16—C12	111.19 (10)	C31B—C36B—H36B	119.5
C18—C16—H16	107.1	C35B—C36B—C31B	121.1
C18—C16—C17	110.66 (15)	C35B—C36B—H36B	119.5
C16—C17—H17A	109.5	C32A—C31A—C36A	118.5 (9)
C16—C17—H17B	109.5	C32A—C31A—C37A	127.4 (9)
C16—C17—H17C	109.5	C36A—C31A—C37A	114.1 (8)
H17A—C17—H17B	109.5	C31A—C32A—H32A	119.1
H17A—C17—H17C	109.5	C31A—C32A—C33A	121.9 (10)
H17B—C17—H17C	109.5	C33A—C32A—H32A	119.1
C16—C18—H18A	109.5	C32A—C33A—H33A	120.8
C16—C18—H18B	109.5	C34A—C33A—C32A	118.4 (10)
C16—C18—H18C	109.5	C34A—C33A—H33A	120.8
H18A—C18—H18B	109.5	C33A—C34A—H34A	120.1
H18A—C18—H18C	109.5	C33A—C34A—C35A	119.7 (10)
H18B—C18—H18C	109.5	C35A—C34A—H34A	120.1
C20—C19—N2	118.87 (9)	C34A—C35A—H35A	119.5
C24—C19—N2	120.13 (9)	C36A—C35A—C34A	121.0 (10)
C24—C19—C20	120.51 (10)	C36A—C35A—H35A	119.5
C19—C20—C25	120.74 (9)	C31A—C36A—H36A	120.0
C21—C20—C19	118.57 (11)	C35A—C36A—C31A	120.0 (10)
C21—C20—C25	120.64 (10)	C35A—C36A—H36A	120.0
C20—C21—H21	119.4	C31A—C37A—H37D	109.5
C22—C21—C20	121.24 (11)	C31A—C37A—H37E	109.5
C22—C21—H21	119.4	C31A—C37A—H37F	109.5
C21—C22—H22	120.1	H37D—C37A—H37E	109.5
C21—C22—C23	119.86 (11)	H37D—C37A—H37F	109.5
C23—C22—H22	120.1	H37E—C37A—H37F	109.5
C22—C23—H23	119.4		
			
N1—C1—C2—C3	−146.50 (10)	C11—C12—C16—C18	−86.69 (15)
N1—C1—C2—C6	37.47 (15)	C12—C7—C8—C9	3.05 (16)
N1—C7—C8—C9	−179.75 (10)	C12—C7—C8—C13	−174.24 (10)
N1—C7—C8—C13	2.96 (16)	C13—C8—C9—C10	175.13 (12)
N1—C7—C12—C11	−178.67 (10)	C19—N2—C1—N1	−0.92 (16)
N1—C7—C12—C16	2.54 (15)	C19—N2—C1—C2	178.39 (9)
N2—C1—C2—C3	34.15 (13)	C19—C20—C21—C22	0.43 (18)
N2—C1—C2—C6	−141.88 (11)	C19—C20—C25—C26	−93.53 (13)
N2—C19—C20—C21	170.13 (10)	C19—C20—C25—C27	142.67 (11)
N2—C19—C20—C25	−12.43 (15)	C19—C24—C28—C29	147.41 (11)
N2—C19—C24—C23	−170.09 (10)	C19—C24—C28—C30	−88.34 (13)
N2—C19—C24—C28	7.60 (15)	C20—C19—C24—C23	1.76 (16)
N3—C5—C6—C2	0.18 (19)	C20—C19—C24—C28	179.45 (10)
C1—N1—C7—C8	65.88 (15)	C20—C21—C22—C23	0.99 (19)
C1—N1—C7—C12	−116.81 (12)	C21—C20—C25—C26	83.85 (14)
C1—N2—C19—C20	103.81 (12)	C21—C20—C25—C27	−39.94 (16)
C1—N2—C19—C24	−84.22 (13)	C21—C22—C23—C24	−1.05 (19)
C1—C2—C3—C4	−175.29 (10)	C22—C23—C24—C19	−0.32 (17)
C1—C2—C6—C5	174.92 (10)	C22—C23—C24—C28	−178.01 (11)
C2—C3—C4—N3	0.11 (19)	C23—C24—C28—C29	−34.95 (16)
C3—C2—C6—C5	−1.16 (16)	C23—C24—C28—C30	89.31 (14)
C4—N3—C5—C6	0.93 (19)	C24—C19—C20—C21	−1.81 (16)
C5—N3—C4—C3	−1.08 (19)	C24—C19—C20—C25	175.62 (10)
C6—C2—C3—C4	1.03 (16)	C25—C20—C21—C22	−177.01 (12)
C7—N1—C1—N2	−178.19 (10)	C37B—C31B—C32B—C33B	178.5
C7—N1—C1—C2	2.53 (16)	C37B—C31B—C36B—C35B	−178.5
C7—C8—C9—C10	−2.29 (18)	C31B—C32B—C33B—C34B	0.2
C7—C8—C13—C14	109.96 (13)	C32B—C31B—C36B—C35B	0.4
C7—C8—C13—C15	−126.43 (12)	C32B—C33B—C34B—C35B	0.0
C7—C12—C16—C17	−142.62 (14)	C33B—C34B—C35B—C36B	0.0
C7—C12—C16—C18	92.07 (14)	C34B—C35B—C36B—C31B	−0.2
C8—C7—C12—C11	−1.39 (16)	C36B—C31B—C32B—C33B	−0.3
C8—C7—C12—C16	179.82 (10)	C31A—C32A—C33A—C34A	−3.4 (17)
C8—C9—C10—C11	−0.1 (2)	C32A—C31A—C36A—C35A	1 (2)
C9—C8—C13—C14	−67.28 (14)	C32A—C33A—C34A—C35A	7 (2)
C9—C8—C13—C15	56.33 (15)	C33A—C34A—C35A—C36A	−7 (3)
C9—C10—C11—C12	1.9 (2)	C34A—C35A—C36A—C31A	2 (2)
C10—C11—C12—C7	−1.16 (18)	C36A—C31A—C32A—C33A	−0.7 (18)
C10—C11—C12—C16	177.65 (11)	C37A—C31A—C32A—C33A	179.2 (14)
C11—C12—C16—C17	38.62 (18)	C37A—C31A—C36A—C35A	−178.7 (15)

### Hydrogen-bond geometry (Å, º)

**Table:**

*D*—H···*A*	*D*—H	H···*A*	*D*···*A*	*D*—H···*A*
N1—H1···N3^i^	0.89 (1)	2.38 (1)	3.118 (1)	141 (1)
C10—H10···N2^ii^	0.95	2.74	3.515 (2)	139
C13—H13···N1	1.00	2.50	2.9794 (15)	109
C16—H16···N1	1.00	2.44	2.8811 (15)	106
C25—H25···N2	1.00	2.42	2.8933 (15)	108
C28—H28···N2	1.00	2.54	2.9140 (15)	102

Symmetry codes: (i) *x*, −*y*+1/2, *z*+1/2; (ii) *x*−1, *y*, *z*.
